# Correlation between *UGT1A1* gene polymorphism and irinotecan chemotherapy in metastatic colorectal cancer: a study from Guangxi Zhuang

**DOI:** 10.1186/s12876-020-01227-w

**Published:** 2020-04-07

**Authors:** Shaojun Chen, Li Hua, Chengjun Feng, Qia Mo, Mengzhuan Wei, Yongqi Shen, Zhan Lin, Guisheng Li, Junyi Xu, Chengxian Guo, Haixin Huang

**Affiliations:** 1 Department of Oncology, the Forth Affiliated Hospital of Guangxi Medical University, No.1 liushi Road, Liuzhou, 545005 Guangxi China; 2 Department of Chemotherapy, Liuzhou Cancer Hospital, Liuzhou, 545006 Guangxi China; 3 Department of Oncology, The Liuzhou Railway Affiliated Hospital of Guangxi Medical University, Liuzhou, 545007 Guangxi China; 4 Department of Oncology, The Yulin First People’s Hospital, Yulin, 537000 Guangxi China; 5 Department of Gastroenterological Surgery, The Fourth Affiliated Hospital of Guangxi Medical University, Liuzhou, 545005 Guangxi China; 6 Clinical Pharmacology Center, The Third Xiangya Hospital of Central South University, Changsha, 410013 Hunan China

**Keywords:** Zhuang of Guangxi, *UTG1A1* gene polymorphisms, Chemotherapy, Irinotecan, Metastatic colorectal cancer

## Abstract

**Background:**

There are obviously ethnic differences between the *UGT1A1* gene polymorphisms. Due to the difference of genetic background and environment, the treatment with colorectal cancer patients of Guangxi Zhuang should not completely follow the Euramerican or Chinese han patients. The study aimed to explore the correlation of *UGT1A1* gene polymorphism of Guangxi Zhuang metastatic colorectal cancer (mCRC) with irinotecan -based chemotherapy, in order to develop an individualized irinotecan regimen for mCRC patients of Guangxi Zhuang.

**Methods:**

From June 2013 and June 2015, a total of 406 patients of Guangxi who were histologically diagnosed as metastatic colorectal cancer with 102 patients of this cohort with three generations of Zhuang, and 86 patients that conformed to inclusion and exclusion criteria were competitively enrolled. The distribution of *UGT1A1* gene polymorphism was analyzed-retrospectively in all patients. Pyrosequencing method was used to detect the UGT1A1*28 and*6 gene polymorphism in the 86 Guangxi Zhuang mCRC patients. After first-line chemotherapy with FOLFIRI regimen, the relationship between gene polymorphism of *UGT1A1* and adverse reactions, and efficacy of Irinotecan were analyzed with χ2 test and Kaplan-Meier method.

**Results:**

UGT1A1*28 wild-type (TA6/6), heterozygous mutant (TA6/7) and homozygous mutant (TA7/7) accounted for 69.8, 30.2 and 0%, respectively. UGT1A1*6 wild type (G/G), heterozygous mutation type (G/A) and homozygous mutant (A/A) accounted for 76.7%, 20.9 and 2.3%, respectively. UGT1A1*28 TA6/7 type could increase the risk of grade 3~4 diarrhea (*p* = 0.027), which did not increase the risk of grade 3~4 neutropenia (*p* = 0.092). UGT1A1*6G/A and A/A type could increase the risk of grade 3~4 diarrhea and neutropenia (*p* = 0.001; *p* = 0.017). After chemotherapy with FOLFIRI, there was no significant difference in response rate (RR) (*p* = 0.729; *p* = 0.745) or in median progression-free survival (mPFS) between the wild-type, mutant treatment of UGT1A1*28 and UGT1A1*6 (7.0 m vs 7.4 m, *p* = 0.427; 6.9 m vs 7.0 m *p* = 0.408).

**Conclusions:**

The distribution of UGT1A1*28 and UGT1A1*6 gene polymorphism in Guangxi Zhuang patients were differed from the existing reported of European people and Chinese Han population. The *UGT1A1* gene polymorphism with irinotecan chemotherapy-associated diarrhea and neutropenia were closely related. There was no significant association between *UGT1A1* gene polymorphism and therapeutic efficacy of irinotecan.

## Background

Colorectal cancer (CRC) is a common gastrointestinal malignancy, with the third highest incidence and fourth highest mortality among malignant tumors worldwide [1, 2]. Since the early symptoms of CRC are obscure, majority of the patients are diagnosed in the intermediate and late stages, and suffer from metastasis at diagnosis, with a 5-year survival rate < 10%. Chemotherapy is the main treatment method for CRC [3–5].

Irinotecan-based chemotherapy is a standard first-line or second-line regimen for the treatment of mCRC. However, irinotecan has dose-limiting toxicities, mainly neutropenia and delayed-onset diarrhea, wherein the incidence of grade 3~4 delayed-onset diarrhea and neutropenia is high. Studies have shown that *UGT1A1* gene polymorphism is differently distributed among different ethnicities, which may lead to different toxicities and efficacies of irinotecan [6]. Even with the same ethnicity, the gene frequency differs in varying geographical regions [7]. Moreover, the frequency of gene mutation differs among various ethnic groups due to different genetic backgrounds. Thus, the therapeutic regimen of irinotecan should be individualized for different ethnic groups, rather than referring to the guidelines of Europe or America for Chinese Han patients. This study aimed to analyze the distribution of *UGT1A1* gene polymorphisms in mCRC patients of Guangxi Zhuang, as well as the correlation of *UGT1A1* gene polymorphisms with the adverse reactions and efficacy of irinotecan chemotherapy, in order to develop an individualized irinotecan regimen for mCRC patients of Guangxi Zhuang.

## Methods

### Patient selection

A total of 406 mCRC patients receiving initial treatment in four centers of Guangxi between June 01, 2013 and June 01, 2015 were selected. After three generations of strict screening, 102 patients were found to be fully compliant with the criteria of Chinese Zhuang, of which 16 were excluded due to severe diseases and incomplete follow-up data. Eligibility criteria included: 1) Patients were diagnosed as mCRC by pathological and imaging results, and did not previously receive chemoradiotherapy; 2) Patients were Guangxi Zhuang for three generations; 3) Patients had measurable lesions by MRI or CT examination; 4) Patients had a ECOG PS ≤1 point, with an expected survival of ≥3 months; 5) Patients did not have significant contraindications to chemotherapy in routine blood, liver and kidney function, ECG and other examinations prior to chemotherapy; 6) Patients had no history of other malignancies; 7) Patients signed informed consent; 8) Patients were routinely followed-up. The selected patients were aged 21~76 years (median age: 56 years), and their clinical data are listed in Table 1. This study was approved by the local ethics committee. Informed consent was obtained from all individual participants included in the study.

**Table 1:** Clinical characteristics of patients with mCRC

Variable	Number	Percentage (%)
Age (years)		
< 60	49	57.0
≥ 60	37	43.0
Gender		
Male	54	62.8
Female	32	37.2
PS score		
0	29	33.7
1	57	66.3
Tumor site		
Colon	36	41.9
Rectum	50	58.1
Number of metastatic sites		
1	51	59.3
≥ 2	35	40.7

### Treatment regimen

#### *UGT1A1* genotyping

Genotyping studies were performed by an independent laboratory (Department of Clinical Pharmacology, Xiangya Hospital, Central South University), and 2 mL peripheral blood was collected in an EDTA containing glass tube about 1 week before the first chemotherapy. Subsequently, genomic DNA was extracted using the phenol-chloroform method, the target gene fragment was amplified using PCR, and the PCR products were sequenced by pyrosequencing. The UGT1A1*28 and *6 polymorphisms were read using the SNP analysis software.

### Chemotherapy regimen

The selected patients received a first-line chemotherapy with irinotecan (CPT-11) + 5-Fluorouracil (5-Fu)/LV (FOLFIRI) regimen. The dosage and administration were as follows: patients were given intravenous infusion of irinotecan (180 mg/m2, 90 mins),d1,intravenous infusion of LV (400 mg/m2),d1, and intravenous injection of 5-Fu (400 mg/m2),d1. Then, the patients were given continuous intravenous injection of 5-Fu (2400 mg/m2),46~48 h. All the treatment were repeated every 2 weeks for 4~6 cycles. The efficacy was evaluated after patients were given at least four cycles of treatment.

### Response and toxicity evaluation

The follow-up ranged from 31~55 months with a median 40 months as of Jan 2018. Four cases were lost to follow-up, with a follow-up rate of 95.56%. The primary endpoint of this study was toxicity, and the secondary endpoint included short-term efficacy and progression-free survival (PFS). The efficacy was evaluated according to the response evaluation criteria in solid tumors, Revised RECIST guidelines (version 1.1) [8]. The adverse reactions were evaluated according to the evaluation and classification of neutropenia, delayed diarrhea in National Cancer Institute NCI-CTC (version 3.0) [9].

### Endpoints and statistical analysis

All SNPs were analyzed for deviation from Hardy-Weinberg equilibrium (HWE),and the genotype and allelic frequencies were calculated. Two-tailed Fisher’s exact test was used to calculate the genotype distribution in controls and patients, and to compare the differences between both groups; χ2 test or Fisher exact probability was used to analyze the adverse reactions and efficacy. Logistic regression was adopted to analyze the relationship between the clinical characteristics of patients and the adverse reactions of irinotecan. Kaplan-Meier method was used to plot the survival curve as well as analyze the relationship between different genotypes and PFS.

## Results

### Distribution of the *UGT1A1* genotype

**Fig. 1:**
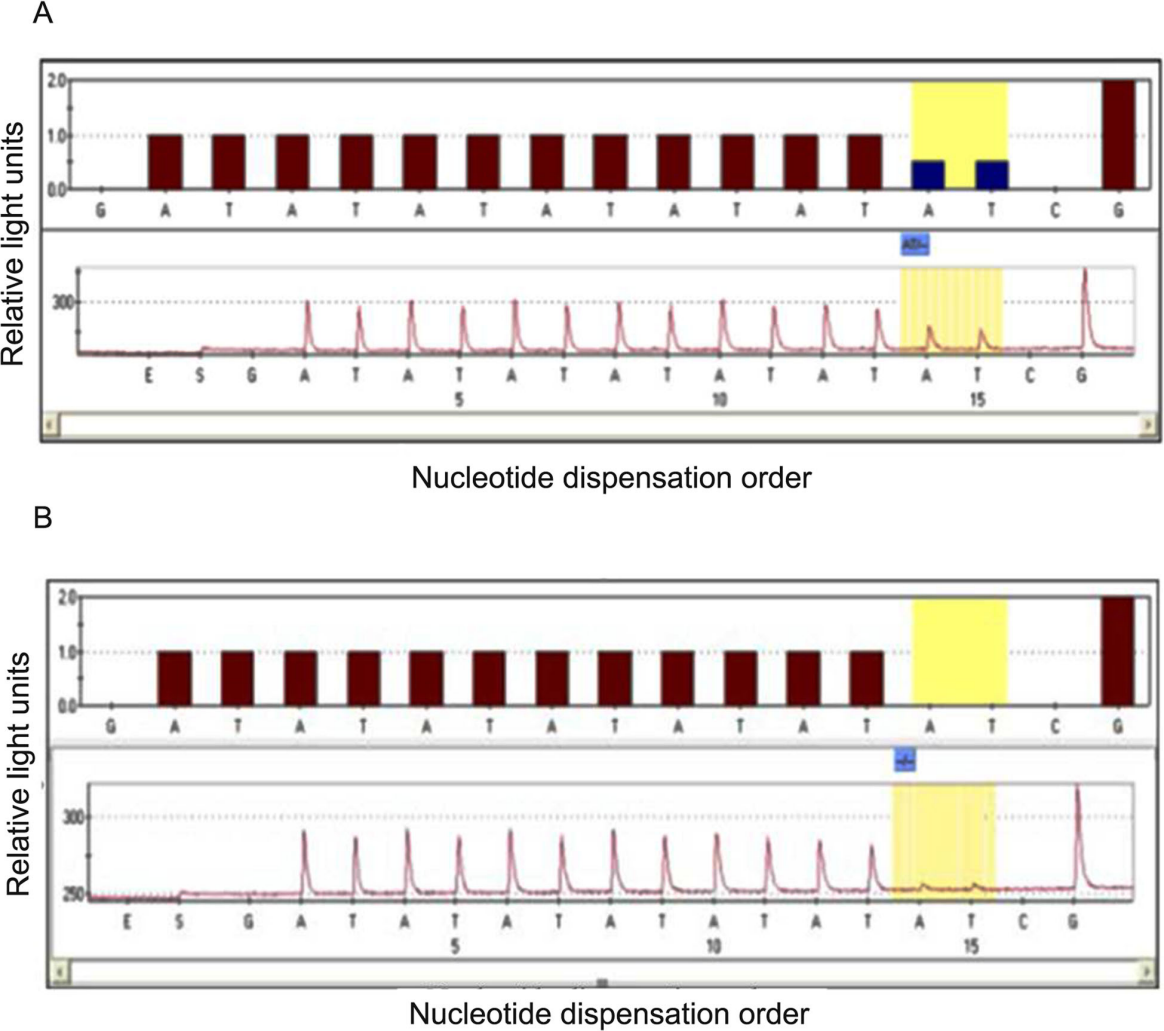
Sequencing results of UGT1A1*28 gene polymorphism. UGT1A1*28 wild-type (**a**), heterozygous-type (**b**)

**Fig. 2:**
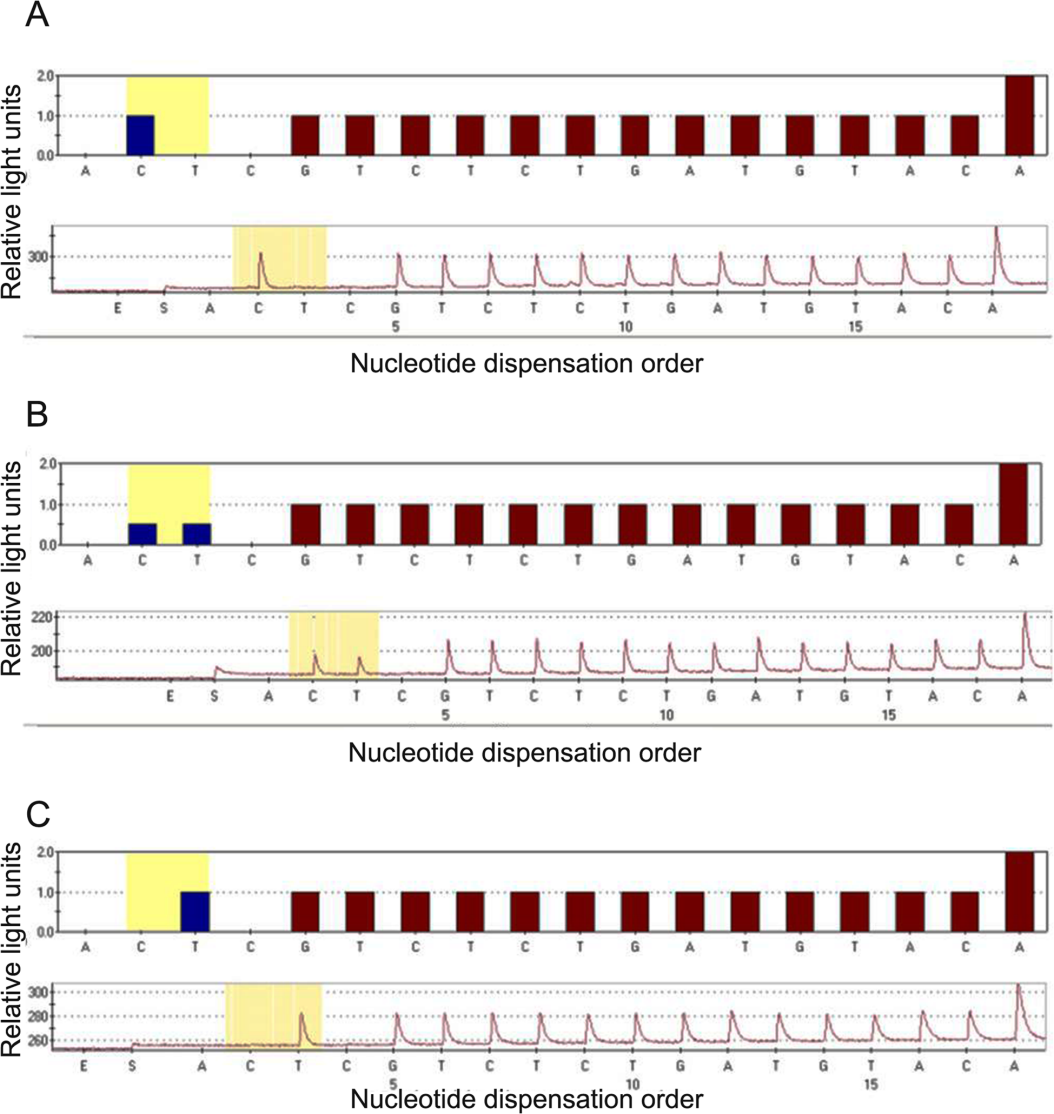
Sequencing results of UGT1A1*6 gene polymorphism. UGT1A1*6 wild-type (**a**), heterozygous–type (**b**), homozygous-type (**c**)

The genotype distribution of *UGT1A1* gene*28 and *6 in 86 patients was consistent with the Hardy-Weinberg equilibrium (HWE) (*p* > 0.1) in population genetics, as shown in Table 2. For the UGT1A1*28, there were 60 cases (69.8%) of wild-type (TA6/6), 26 cases (30.2%) of heterozygous–type (TA6/7), and no case of homozygous-type (TA7/7). The sequencing results are shown in Fig. 1. For the UGT1A1*6, there were 66 cases (76.7%) of wild-type (G/G), 18 cases (20.9%) of heterozygous–type (G/A) and two cases (2.3%) of homozygous-type (A/A). The sequencing results are shown in Fig. 2.

**Table 2:** Distribution of UGT1A1 genotype in Guangxi Zhuang

Variable	Number (%)	Allelic frequencies	H-W equilibrium p
UGT1A1*28			
Wild TA6/6	60 (69.8)	TA6 0.849	0.306
Heterozygous TA6/7	26 (30.2)	TA7 0.151	
Homozygous TA7/7	0 (0)		
UGT1A1*6			
Wild (G/G)	66 (76.7)	G 0.872	0.834
Heterozygous (G/A)	18 (20.9)	A 0.128	
Homozygous (A/A)	2 (2.3)

### Toxicity to chemotherapy based on the *UGT1A1* genotype

**Fig. 3:**
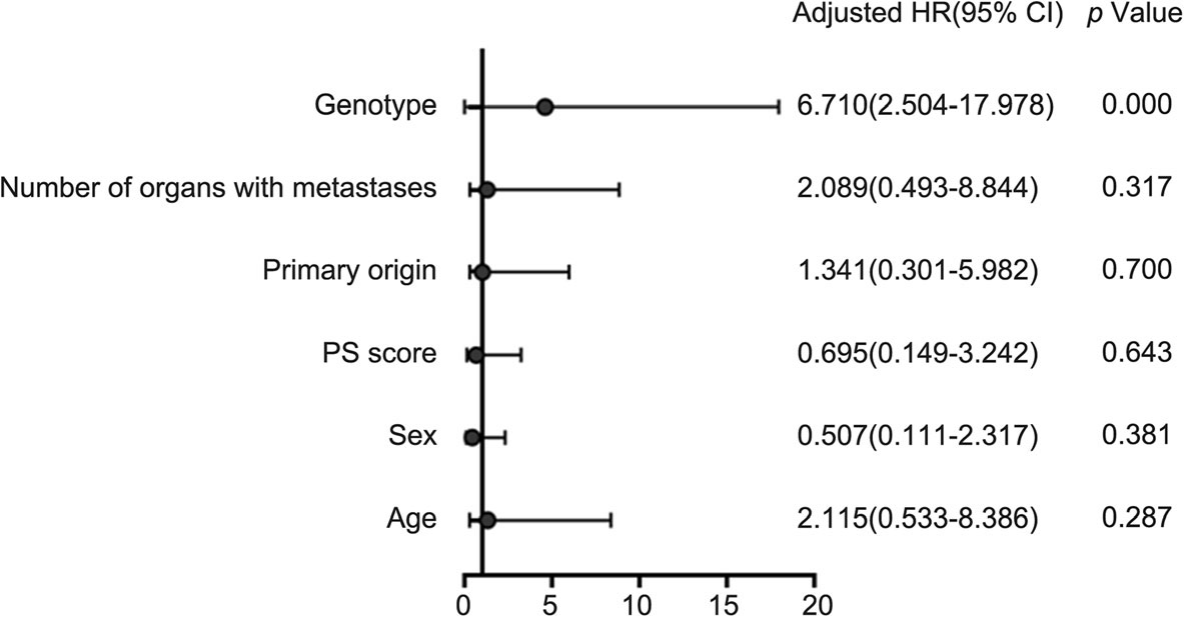
Multiple factors analysis of delayed diarrhea by Logistic regression

**Fig. 4:**
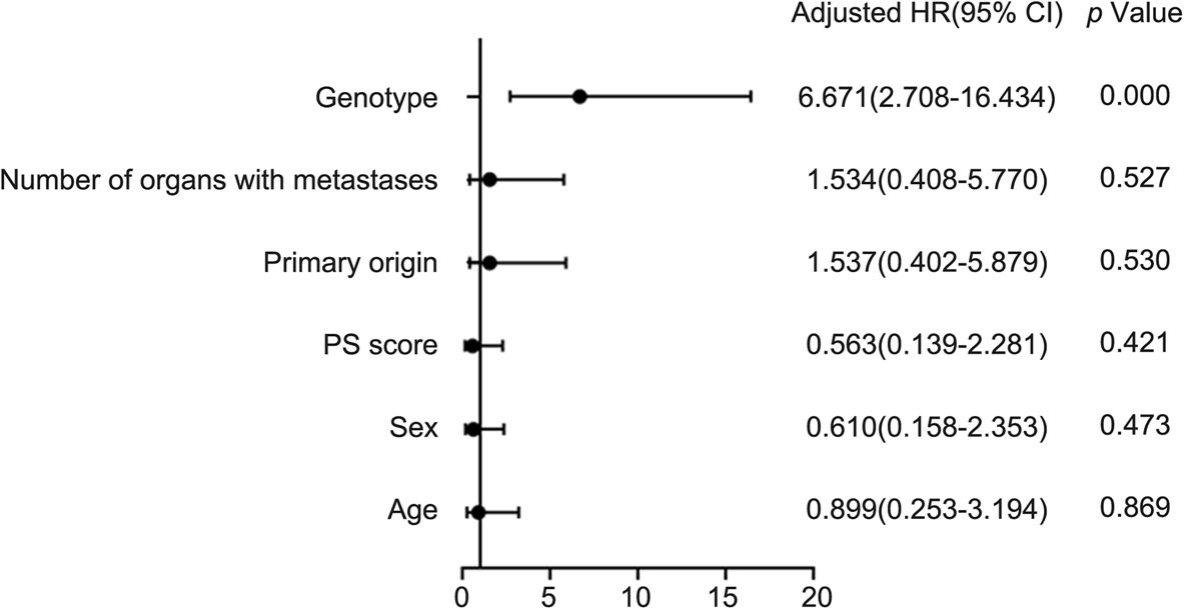
Multiple factors analysis of neutropenia by Logistic regression

Among the 86 patients, there were 60 cases of UGT1A1*28 TA6/6, of which seven cases (11.7%) experienced grade 3~4 delayed-onset diarrhea and nine cases (15.0%) had grade 3~4 neutropenia. Among the 26 cases of UGT1A1*28 TA6/7, nine cases (34.6%) experienced grade 3~4 delayed-onset diarrhea and eight cases (30.8%) had grade 3~4 neutropenia. Statistical analysis revealed that UGT1A1*28 TA6/7 patients had a higher risk of grade 3~4 delayed-onset diarrhea as compared to UGT1A1*28 TA6/6 patients, and the difference was statistically significant (χ2 = 4.884, *p* = 0.027). Meanwhile, UGT1A1*28 TA6/7 was unlikely to increase the risk of grade 3~4 neutropenia (χ2 = 2.844, *p* = 0.092). Among the 86 patients, there were 66 cases of UGT1A1*6 G/G, of which six cases (9.1%) experienced grade 3~4 delayed-onset diarrhea, and 10 cases (15.2%) had grade 3~4 neutropenia. Among the 20 cases of UGT1A1*6 G/A + A/A, nine cases (45.0%) had grade 3~4 delayed-onset diarrhea and eight cases (40.0%) experienced grade 3~4 neutropenia. Statistical analysis showed that UGT1A1*6 G/A + A/A patients had a higher risk of grade 3~4 diarrhea as compared to G/G patients, with statistically significant difference (χ2 = 11.364, *p* = 0.001), and also a higher risk of grade 3~4 neutropenia as compared to G/G patients (χ2 = 5.727, *p* = 0.017). In this study, no patient suffered from adverse event-related death. (Table 3). The delayed diarrhea and neutropenia was not associated with gender, age, PS score, primary lesion and the number of metastatic organs(*p* > 0.05) (Table 4), but associated with genotype((Table 5, Fig. 3; Table 6, Fig. 4). The probability of occurrence of grade 3~4 delayed diarrhea and neutropenia in wild-type patients was significantly lower than that in single-point and double-point mutants (χ2 = 8.802, *p* = 0.005, χ2 = 23.171, *p* = 0.000). **(**Tables 7 and 8).

**Table 3:** Toxicity and UGT1A1 status (%)

Toxicity	UGT1A1*28		*P*	UGT1A1*6		*P*
	TA6/6 Number (%)	TA6/7 Number (%)		G/G Number (%)	G/A + A/A Number (%)	
Diarrhea						
0 grade	41 (68.3)	10 (38.5)	0.027	45 (68.2)	7 (35.0)	0.001
1–2 grade	12 (20.0)	7 (26.9)		15 (22.7)	4 (20.0)	
3–4 grade	7 (11.7)	9 (34.6)		6 (9.1)	9 (45.0)	
Neutropenia						
0 grade	33 (55.0)	10 (38.4)	0.092	38 (57.6)	5 (25.0)	0.017
1–2 grade	18 (30.0)	8 (30.8)		18 (27.3)	7 (35.0)	
3–4 grade	9 (15.0)	8 (30.8)		10 (15.2)	8 (40.0)

**Table 4:** Toxicity and clinical characteristics

Clinical feature	Diarrhea			*P*	Neutropenia			*P*
	0 grade	1–2 grade	3–4 grade		0 grade	1–2 grade	3–4 grade	
Age (years)								
< 60	31	12	6	0.332	28	11	10	0.235
≥ 60	21	7	9		15	14	8	
Gender								
Male	32	12	10	0.936	27	15	12	0.905
Female	20	7	5		16	10	6	
PS score								
0	17	7	5	0.947	13	9	7	0.776
1	35	12	10		30	16	11	
Tumor site								
Colon	23	8	5	0.753	18	11	7	0.945
Rectum	29	11	10		25	14	11	
N of metastatic organs								
1	31	12	8	0.843	25	15	11	0.974
2	21	7	7		18	10	7

**Table 5:** Multivariate analysis ofthe logistic risk ratio model for Delayed diarrhea

Delayed diarrhea	β	Sig	Exp(B)	95% CI	
				Lower Bound	Upper Bound
Age	0.749	0.287	2.115	0.533	8.386
Sex	−0.679	0.381	0.507	0.111	2.386
PS scores	−0.364	0.643	0.695	0.149	3.242
Primary origin	0.294	0.700	1.341	0.301	5.982
Number of organs with metastases	0.736	0.317	2.089	0.493	8.844
genotypa	1.904	0.000	6.710	2.504	17.978
constant	−5.337	0.000	0.005

**Table 6:** Multivariate analysis of logistic risk ratio model for neutropenia

neutropenia	β	Sig	Exp(B)	95% CI	
				Lower Bound	Upper Bound
Age	−0.107	0.869	0.899	0.253	3.194
Sex	−0.495	0.473	0.610	0.158	2.353
PS scores	−0.574	0.421	0.563	0.139	2.281
Primary origin	0.430	0.530	1.537	0.402	5.879
Number of organs with metastases	0.428	0.527	1.534	0.408	5.770
genotypa	1.898	0.000	6.671	2.708	16.434
constant	−4.362	0.000	0.013

**Table 7:** Delayed diarrheacompared withUGT1A1wild-type、single-point and double-point mutants (%)

Genotype	Total n	Delayed diarrhea n(%)		X^2^	*P*
UGT1A1*28		0~II	III~IV	6.308	0.017
6/6	60	53 (88.3)	7 (11.7)		
6/7	26	17 (65.4)	9 (34.6)		
UGT1A1*6				13.745	0.001
G/G	66	60 (90.9)	6 (10.0)		
G/A + A/A	20	11(55.0)	9 (24.3)		
Numbers of mutational alleles				8.802	0.005
Double single type	53	50 (94.3)	3 (5.7)		
Single variant	22	17(77.3)	5 (22.7)		
Double variant	11	7 (63.6)	4 (36.3)

**Table 8:** Neutropenia compared withUGT1A1wild-type、single-point and double-point mutants (%)

Genotype	Total n	neutropenia n(%)		X^2^	*P*
UGT1A1*28		0~II	III~IV	2.844	0.139
6/6	60	51 (85.0)	9 (15.0)		
6/7	26	18 (69.2)	8 (30.8)		
UGT1A1*6				5.727	0.027
G/G	66	56 (84.8)	10 (15.2)		
G/A + A/A	20	12 (60.0)	8 (40.0)		
Numbers of mutational alleles				23.171	0.000
Double single type	53	50 (94.3)	3 (5.7)		
Single variant	22	13 (59.1)	9 (40.9)		
Double variant	11	4 (36.4)	7 (63.6)		

### Response to chemotherapy based on the *UGT1A1* genotype

**Fig. 5:**
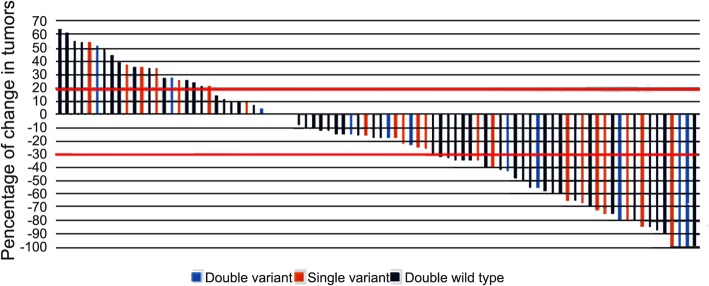
Best percentage change of response to chemotherapy from baseline with *UGT1A1* genotype

Among the 86 UGT1A1*28 patients, there were two cases of CR (7.7%) (both of TA6/7), 32 cases of PR [including 23 TA6/6 cases (38.3%) and nine TA6/7 cases (34.6%)], 30 cases of SD [including 21 TA6/6 cases (35.0%) and nine TA6/7 cases (34.6%)], and 22 cases of PD [including 16 TA6/6 cases (26.7%) and six TA6/7 cases (23.1%)] (Fig. 5). Among the 86 UGT1A1*6 patients, there were two cases of CR [one G/G case (1.5%) and one G/A + A/A case (5.0%)], 34 cases of PR [including 26 G/G cases (39.4%) and eight G/A + A/A cases (40.0%)], 28 cases of SD [including 22 G/G cases (33.3%) and six G/A + A/A cases (30%)], and 22 cases of PD [including 17 G/G cases (25.8%) and five G/A + A/A cases (25%)] (Tables 4 and 5). Of these, the ORRs of UGT1A1*28 TA6/6 and TA6/7 patients after chemotherapy were 38.3 and 42.3% (χ2 = 0.120, *p* = 0.729), respectively, while those of UGT1A1*6 G/G and G/A + A/A patients were 40.9 and 45.0% (χ2 = 0.106, *p* = 0.745), respectively. (Tables 9 and 10).

**Table 9:** Response to treatment and UGT1A1 status (%)

Effect	UGT1A1*28		*P*	UGT1A1*6		*P*
	TA6/6 Number (%)	TA6/7 Number (%)		G/G Number (%)	G/A + A/A Number (%)	
CR	0 (0)	2 (7.7)	0.729	1 (1.5)	1 (5.0)	0.745
PR	23 (38.3)	9 (34.6)		26 (39.4)	8 (40.0)	
SD	21 (35.0)	9 (34.6)		22 (33.3)	6 (30.0)	
PD	16 (26.7)	6 (23.1)		17 (25.8)	5 (25.0)

**Table 10:** Response compared withUGT1A1wild-type、single-point and double-point mutants (%)

Genotype	Total n	ORR % (n)	X^2^	*P*	DCR % (n)	X^2^	*P*
UGT1A1*28			0.120	0.812		0.123	0.794
6/6	60	38.3 (23)			73.3 (44)		
6/7	26	42.3 (11)			76.9 (20)		
UGT1A1*6			0.106	0.799		0.005	1.000
G/G	66	40.9 (27)			74.2 (49)		
G/A + A/A	20	45.0 (9)			75.0 (15)		
Numbers of mutational alleles			0.069	1.000		0.329	0.879
Double single type	53	41.5 (22)			75.5 (40)		
Single variant	22	40.9(9)			72.7 (16)		
Double variant	11	44.5 (5)			81.8 (9)		

### Progression-free survival based on the *UGT1A1* genotype

**Fig. 6:**
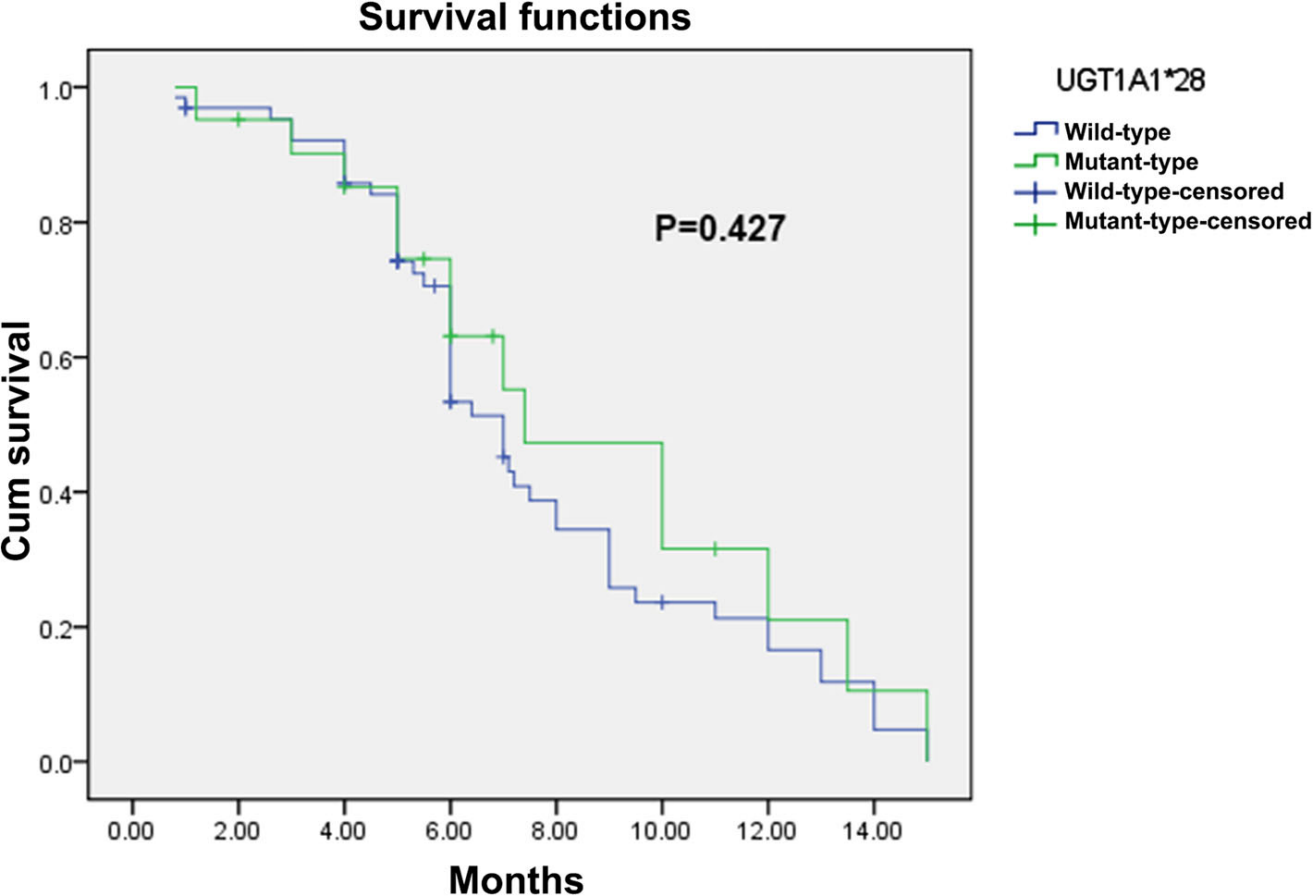
Progression-Free Survival of patients with wild-type *UGT1A1* * 28 and mutant-type *UGT1A1* * 28

**Fig. 7:**
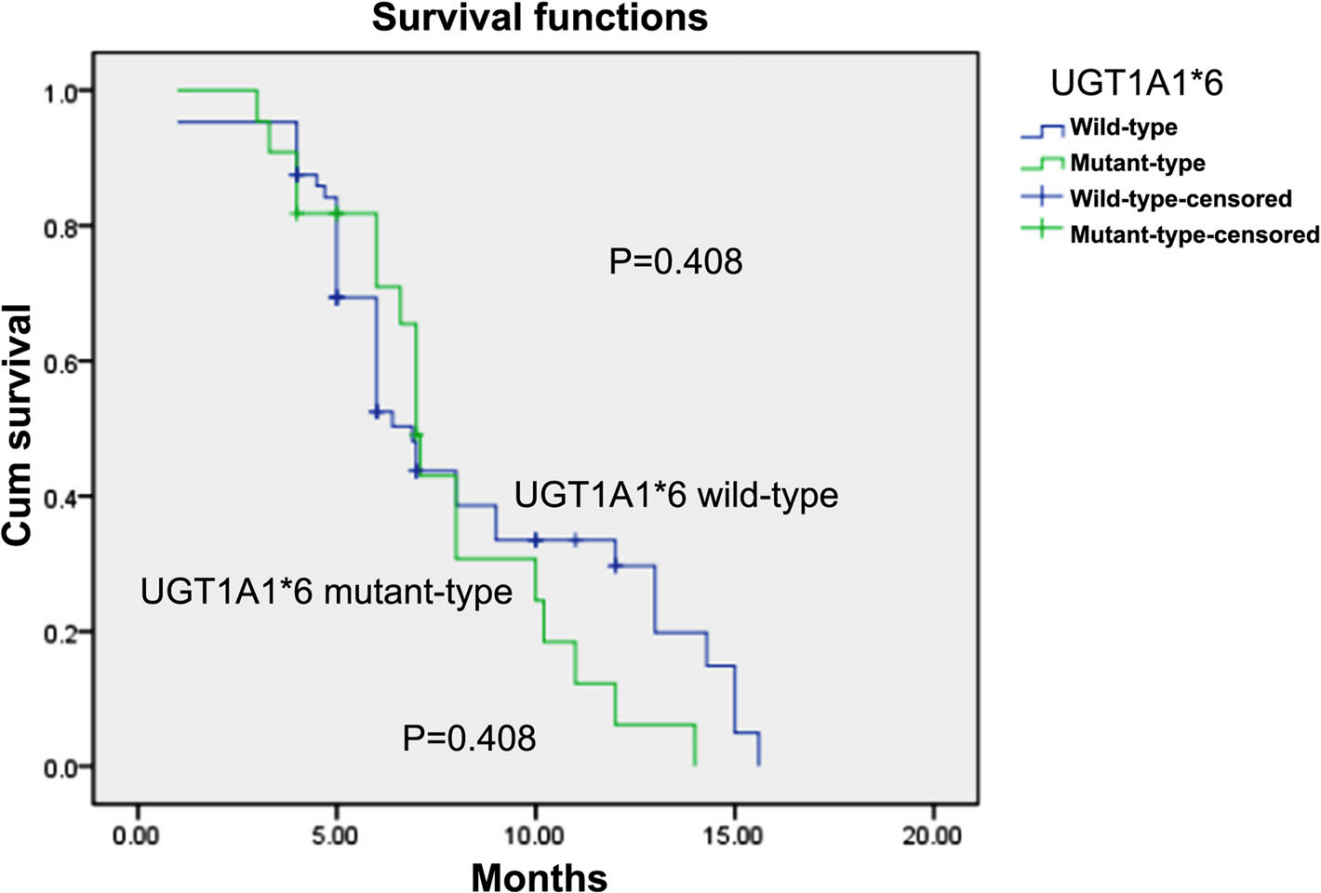
Progression-Free Survival of patients with wild-type *UGT1A1* * 6 and mutant-type *UGT1A1* * 6

Patients were followed-up until June 01, 2017. The median PFS of UGT1A1*28 TA6/6 and TA6/7 patients were 7.0 and 7.4 months, respectively, with statistically insignificant difference (*p* = 0.427), and those of UGT1A1*6 G/G and G/A + A/A patients were 6.9 and 7.0 months, respectively, with statistically insignificant difference (*p* = 0.408). The survival curves plotted using the Kaplan-Meier method are shown in Figs. 6 and 7.

### Distribution of the *UGT1A1* genotype with irinotecan dose reduction

Among the 86 patients, irinotecan dose was reduced in 15 patients, including six cases of wild-type (TA6/6) and nine cases of mutant-type (TA6/7) UGT1A1*28, and seven cases of wild-type (G/G) and eight cases of mutant-type (G/A) UGT1A1*6. This indicated that a higher ratio of patients with mutant-type UGT1A1*28 and UGT1A1*6 required irinotecan dose reduction as compared to patients with wild-type UGT1A1*28 and UGT1A1*6 (χ2 = 6.019, *p* = 0.014; χ2 = 7.281, *p* = 0.007) (Table 11). During subsequent chemotherapy, the irinotecan dose was reduced by about 20%. Fifteen patients could tolerate the reduced dose of irinotecan, and did not require irinotecan dose reduction to the next gradient.

**Table 11:** Correlation between UGT1A1 genetic polymorphisms and irinotecan dose reduction in patients with CRC

Irinotecan dose reduction	UGT1A1*28		*P*	UGT1A1*6		*P*
	TA6/6 Number (%)	TA6/7 + TA7/7 Number (%)		G/G Number (%)	G/A Number (%)	
Yes	6 (10.0)	9 (45.0)	0.014	7 (10.6)	8 (40.0)	0.007
No	54 (90.0)	11 (55.0)		59 (89.4)	12 (60.0)	

### Toxicity of irinotecan dose reduction based on the *UGT1A1* genotype

After the irinotecan dose was reduced, the incidences of grade 3~4 diarrhea and grade 3~4 neutropenia were significantly decreased in both the dose reduction group and non-dose reduction group for patients with UGT1A1*28 (*p* = 1.000; *p* = 0.613) and UGT1A1*6 (*p* = 0.442; *p* = 0.139) in Table 12.

**Table 12:** Toxicity with irinotecan dose reduction and UGT1A1 status (%)

Toxicity	UGT1A1*28		UGT1A1*6	
	Dose reduction	Non-dose reduction	Dose reduction	Non-dose reduction
Diarrhea				
0 grade	6 (40.0)	47 (66.2)	8 (53.3)	45 (63.4)
1–2 grade	9 (60.0)	21 (29.6)	6 (40.0)	24 (33.8)
3–4 grade	0 (0.0)	3 (4.2)	1 (6.7)	2 (2.8)
Neutropenia				
0 grade	9 (60.0)	45 (63.4)	9 (60.0)	48 (67.6)
1–2 grade	5 (33.3)	21 (29.6)	4 (26.7)	21 (29.6)
3–4 grade	1 (6.7)	5 (7.0)	2 (13.3)	2 (2.8)

### Efficacy of irinotecan dose reduction based on the *UGT1A1* genotype

**Fig. 8:**
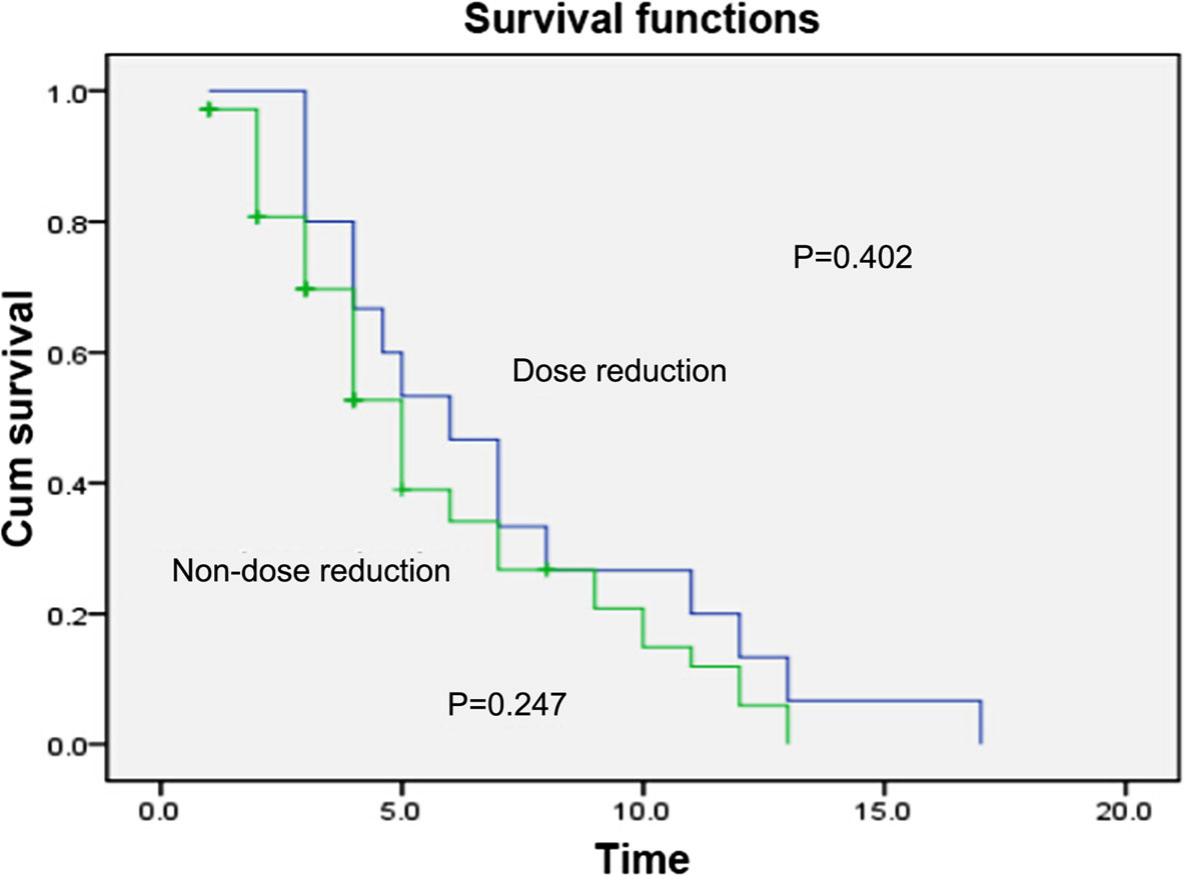
Comparison of long-term effects between the dose reduction group and non-reduction group in patients with *UGT1A1* * 28 gene

**Fig. 9:**
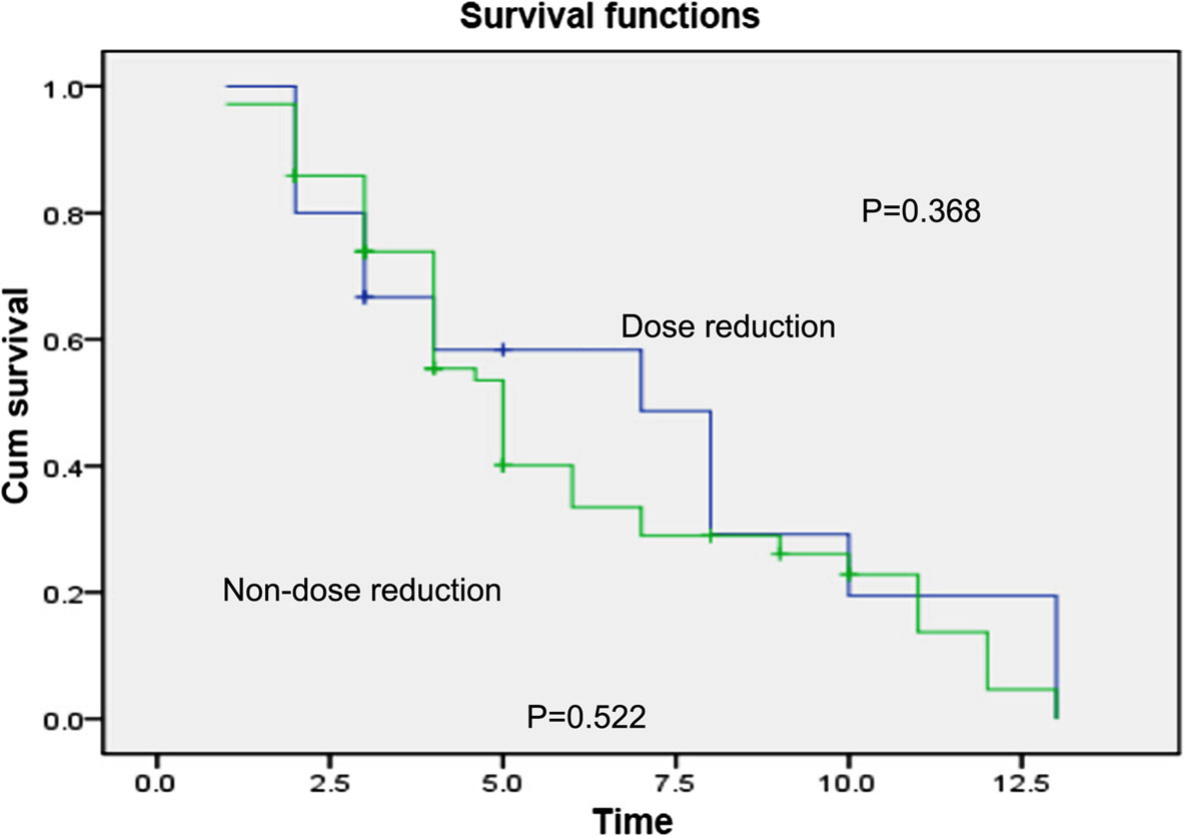
Comparison of long-term effect between the dose reduction group and non-reduction group in patients with *UGT1A1* * 6 gene

In patients with UGT1A1*28 and UGT1A1*6 genes, there was insignificant difference in the short-term effect between the dose reduction group and non-dose reduction group (*p* = 0.402, *p* = 0.368) **(**Figs. 8 and 9**)**. Also, there was insignificant difference in the median PFS between the dose reduction group and non-dose reduction group (χ2 = 1.946, *p* = 0.378; χ2 = 1.895, *p* = 0.388) (Table 13).

**Table 13:** Comparison of short-term effects between the irinotecan dose reduction group and non-dose reduction group

Effect	UGT1A1*28		*p*	UGT1A1*6		*p*
	Dose reduction	Non-dose reduction		Dose reduction	Non-dose reduction	
PR	2 (13.3)	11 (15.5)	0.378	4 (26.7)	9 (12.7)	0.388
SD	9 (60.0)	29 (40.8)		6 (40.0)	33 (46.5)	
PD	4 (26.7)	31 (43.7)		5 (33.3)	29 (40.8)	

## Discussion

Irinotecan is a semi-synthetic derivative of natural camptothecin, and has been widely used in the treatment of solid tumors such as gastric cancer, colorectal cancer (CRC), lung cancer, etc. A combination of irinotecan and fluorouracil is a standard first-line regimen for advanced CRC, especially advanced CRC with rapid progression, with an efficacy rate of up to 40% [10–12]. However, this regimen has two major adverse reactions of delayed-onset diarrhea and neutropenia, where the incidences of grade 3~4 neutropenia and severe diarrhea are 45% and 20~40% [13], respectively, which limits its clinical application and exhibits inter-individual variations.

Irinotecan is hydrolyzed in vivo by carboxylesterase (CE) into an active metabolite 7-ethyl-10-hydroxycamptothecin (SN-38). The latter is a topoisomerase I inhibitor that inhibits repair of broken single-stranded DNA, disrupts DNA replication and transcription, and exerts cytotoxic effects. SN-38 is inactivated by *UGT1A1* as glucuronic acid product SN-38G, which is excreted into the intestine through the bile and is transformed into SN-38 by the intestinal bacterial β-glucuronidase, thereby inducing mucosal injury and delayed-onset diarrhea. *UGT1A1* enzyme in the intestine can re-catalyze SN-38 for SN-38G detoxification. Therefore, the adverse reactions of irinotecan are related to its main drug-metabolizing enzyme *UGT1A1*, whose activity is affected by polymorphism. UGT1A1*28 polymorphism and irinotecan-related adverse reactions have been reported in many studies, but the correlation between UGT1A1*6 polymorphism and adverse reactions of irinotecan remains unclear. Several studies, with conflicting results, have indicated that the *UGT1A1* polymorphism has insufficient sensitivity and specificity in predicting the adverse effects of irinotecan. The results of different ethnic groups in the same race are rarely reported. In addition, the role of *UGT1A1* polymorphism in predicting the toxicity in patients receiving different doses of irinotecan is clinically controversial.

Due to differences in genetic background, different *UGT1A1* mutation sites exist among various ethnic groups, which is responsible for different incidences of toxicity between different populations in eastern and western countries receiving irinotecan treatment. Studies on the distribution of *UGT1A1* *28 and *6 polymorphisms have demonstrated that *UGT1A1* * 28 homozygous mutant TA7/7 accounts for 10~15% and 12~27% in the Caucasian and African populations, respectively [14, 15], while its mutation rate is only 1.2~4.7% in Asian population [16, 17]. Nakaura et al. [18] showed that the wild-type UGT1A1*28 accounted for 46 and 76% in the Caucasian and Asian populations, respectively, indicating significant inter-ethnic differences. Etienne-Grimald et al. [19] concluded that UGT1A1*28 had the highest expression frequency in Americans and Caucasians, accounting for 38~45% and 29~39%, respectively, while a mutation frequency of about 15~18% in Asians, of which the homozygous mutation type accounted for 3%. Zhang et al. [20] investigated the distribution of UGT1A1*28 polymorphism in 517 Han patients, and found that UGT1A1*28 TA6/6, TA7/6 and TA7/7 accounted for 77.2, 22 and 0.8%, respectively. In this study, the distribution frequencies of wild-type and heterozygous-type UGT1A1*28 were 69.8 and 30.2%, respectively, in 86 patients in Guangxi Zhuang, with no homozygous-type case, which indicated that the homozygous mutation rate was further reduced as compared to the Asian Han ethnicity.

UGT1A1*6 is a unique *UGT1A1* mutation type in Asian populations. The frequencies of UGT1A1*6 homozygous mutant A/A in Koreans and Japanese were reported to be 7 and 4%, respectively, while the frequencies of UGT1A1*6 G/G, G/A and A/A in Han Chinese were 66.9, 29.3 and 3.8%, respectively [20]. This study revealed that the frequencies of wild-type, heterozygous-type and homozygous-type UGT1A1*6 in Guangxi Zhuang were 76.7, 20.9 and 2.3%, respectively, which differed from the reported distribution frequencies of genetic polymorphisms in Europeans and Americans, wherein the mutation rate was slightly lower than that in Chinese Han and other Asians. However, this may be due to a small sample size. Equilibrium test of UGT1A1*28 and UGT1A1*6 allelic frequencies in this study revealed that the samples were in accordance with the Hardy-Weinberg equilibrium, derived from a larger and randomized marriage-balanced population, and was representative.

Clinical studies have shown that Caucasians with UGT1A1*28 gene mutants (TA6/7 and TA7/7) have a higher risk of severe granulocytopenia and diarrhea than those with wild-type UGT1A1*28 (TA6 /6) after receiving irinotecan [21]. In our study, Guangxi Zhuang patients with UGT1A1*28 mutations showed a higher risk of 3~4 grade delayed-onset diarrhea as compared to those with wild-type UGT1A1*28 (30.8% vs. 11.7%, *p* = 0.044), but did not have a higher risk of 3~4 grade neutropenia (34.6% vs. 15.0%, *p* = 0.112), which was consistent with other domestic reports [22]. Moreover, Guangxi Zhuang patients with UGT1A1*6 mutations had a higher risk of 3~4 grade delayed-onset diarrhea after receiving irinotecan (45% vs. 9.1%, *p* = 0.001), and an increased risk of 3~4 grade neutropenia (40% vs. 15.2%, *p* = 0.017), which was consistent with numerous clinical studies in Japan [23–25]. The correlation between *UGT1A1* genotype and adverse reactions of irinotecan remains controversial. For the effect of UGT1A1* 28 mutant on delayed-onset diarrhea and 3~4 grade granulocytopenia, only one adverse reaction was mentioned in some studies. For example, Miyata et al. only compared the risk of neutropenia, but not the risk of delayed-onset diarrhea [26]. Also, the predictive effects were refuted in some studies, but supported in other studies. Some studies supported the predictive effect on 3~4 grade neutropenia but refuted the risk of delayed-onset diarrhea [17], while others supported the occurrence of delayed-onset diarrhea but refuted the risk of 3~4 grade neutropenia [22]. In addition to the racial differences, we believe it is also related to the dose of irinotecan. In many studies, Caucasians were enrolled and a larger initial dose of irinotecan may have been selected, but the dose of irinotecan was not investigated. Previous studies revealed that UGT1A1*28 and UGT1A1*6 have a predictive effect on the adverse reactions of intermediate or high dose of irinotecan, but not on a low dose of irinotecan [27]. In this study, the homozygous and heterozygous mutations of *UGT1A1* showed a predictive effect when the dose of irinotecan was reduced. For UGT1A1*28 and UGT1A1*6, the incidence of 3~4 grade neutropenia and 3~4 grade diarrhea significantly declined in both the dose reduction and non-reduction groups.

The correlation between *UGT1A1* gene polymorphism and efficacy of irinotecan chemotherapy remains inconclusive. Toffoli et al. [28] believed that the efficacy was better in patients with mutant UGT1A1*28 as compared to those with wild-type UGT1A1*28. However, others believed that the efficacy was better in wild-type patients as compared to mutant patients [29], or the *UGT1A1* gene polymorphism was not correlated with the efficacy [30]. The relationship between *UGT1A1* gene polymorphism and adverse reactions of chemotherapy has been reported in several studies, while the efficacy of chemotherapy has been rarely studied. This study showed that the polymorphism of UGT1A1*28 and UGT1A1*6 genes in Guangxi Zhuang patients with CRC was not significantly correlated with the RR and median PFS of irinotecan chemotherapy. Nevertheless, the correlation between *UGT1A1* polymorphism and efficacy of irinotecan chemotherapy needs further verification with a large sample size study.

The above results may be affected by differences in population, drug dose, and a small sample size. Whether the gene polymorphism distribution and inter-individual variations in response to irinotecan in Chinese Zhuang are different from that in Han as well as European and American populations remains unknown. The sample size should be increased and the follow-up survival should be analyzed based on the gene distribution characteristics of Chinese and combined with the drug dose, in order to achieve the goal of individualization.

## Conclusions

The frequency of gene mutation differs among various ethnic groups due to different genetic backgrounds. In our study, we found the distribution frequencies of wild-type, heterozygous-type and homozygous-type *UGT1A1* in Guangxi Zhuang patients of metastatic colorectal cancer differed from the reported distribution frequencies of genetic polymorphisms in European patients people and Chinese Han patients. The *UGT1A1* gene polymorphism with irinotecan chemotherapy-associated diarrhea and neutropenia were closely related. Guangxi Zhuang patients of metastatic colorectal cancer with UGT1A1*28 mutations showed a higher risk of 3~4 grade diarrhea as compared to those with wild-type UGT1A1*28 which did not increase the risk of grade 3~4 neutropenia. Moreover, Guangxi Zhuang patients with UGT1A1*6 mutations had a higher risk of 3~4 grade diarrhea and neutropenia those with wild-type. After chemotherapy with FOLFIRI, there was no significant difference in response rate and the median progression-free survival between the wild-type, mutant treatment of *UGT1A1* * 28 and *UGT1A1* * 6. The above results may be affected by differences in population, or drug dose. The follow-up survival should be analyzed based on the gene distribution characteristics of Chinese and combined with the drug dose, in order to achieve the goal of individualization.

## Data Availability

The data sets used and/or analyzed during the current study are available from the corresponding author on reasonable request.

## Abbreviations

CE: Carboxylesterase
CRC: Colorectal cancer
HWE: Hardy-Weinberg equilibrium
mCRC: Metastatic colorectal cancer
mPFS: Median progression-free survival
RR: Response rate
*UGT1A1*: Uridine diphosphate glucuronosyl transferase 1A1
